# How COVID-19 will boost remote exercise-based treatment in Parkinson’s disease: a narrative review

**DOI:** 10.1038/s41531-021-00160-3

**Published:** 2021-03-08

**Authors:** Agnes Langer, Lucia Gassner, Anna Flotz, Sebastian Hasenauer, Jakob Gruber, Laurenz Wizany, Rochus Pokan, Walter Maetzler, Heidemarie Zach

**Affiliations:** 1 Department of Neurology, Medical University of Vienna, Vienna, Austria; 2 Department of Sport Physiology, Institute of Sports Sciences, University of Vienna, Vienna, Austria; 3 School of Engineering, RMIT University, Melbourne, VIC Australia; 4 Department of Neurology, University Medical Centre Schleswig-Holstein, Kiel University, Kiel, Germany

**Keywords:** Parkinson's disease, Rehabilitation, Quality of life

## Abstract

The lack of physical exercise during the COVID-19 pandemic-related quarantine measures is challenging, especially for patients with Parkinson’s disease (PD). Without regular exercise not only patients, but also nursing staff and physicians soon noticed a deterioration of motor and non-motor symptoms. Reduced functional mobility, increased falls, increased frailty, and decreased quality of life were identified as consequences of increased sedentary behavior. This work overviews the current literature on problems of supplying conventional physiotherapy and the potential of telerehabilitation, allied health services, and patient-initiated exercise for PD patients during the COVID-19 period. We discuss recent studies on approaches that can improve remote provision of exercise to patients, including telerehabilitation, motivational tools, apps, exergaming, and virtual reality (VR) exercise. Additionally, we provide a case report about a 69-year-old PD patient who took part in a 12-week guided climbing course for PD patients prior to the pandemic and found a solution to continue her climbing training independently with an outdoor rope ladder. This case can serve as a best practice example for non-instructed, creative, and patient-initiated exercise in the domestic environment in difficult times, as are the current. Overall, many recent studies on telemedicine, telerehabilitation, and patient-initiated exercises have been published, giving rise to optimism that facilitating remote exercise can help PD patients maintain physical mobility and emotional well-being, even in phases such as the COVID-19 pandemic. The pandemic itself may even boost the need to establish comprehensive and easy-to-do telerehabilitation programs.

## Introduction

The global COVID-19 pandemic, caused by the coronavirus SARS-CoV-2, poses a high risk on old and chronically ill patients, including patients with Parkinson’s disease (PD)^1–3^. Next to devastating direct consequences caused by the acute illness, COVID-19 also poses risks of long-term effects on PD patients^4,5^. The worldwide pandemic forced countries all over the globe to implement severe quarantine measures. Not only social and public life is reduced to a minimum, but healthcare services have also been cut down considerably. Physiotherapy was among the canceled services, with grave consequences for chronically ill patients^6–10^. Since physical therapy is the basic pillar of their overall therapy, its interruption severely affected PD patients. When the COVID-19 pandemic forced public and private fitness providers alike to close their services abruptly, unable to provide information on reopening dates etc, patients and caregivers were left with the frustrating reality of losing a highly relevant therapeutic strategy^11–13^. This narrative review aims at presenting and discussing the effects of the forced absence of physical therapy in PD and their specific countermeasures. We summarize what is known about exercise-based treatment in PD, and how discontinuity brought about by the COVID-19-lockdown affects PD patients. We then describe in more detail the status of international recommendations on how to continue physiotherapy during COVID-19 and the current state of telerehabilitation, virtual reality (VR), tools for keeping motivation for physical activity, and exergaming, for patients with PD. At last, we present one patient and her unconventional individual solution to the lack of public climbing classes to add empirical evidence for the relevance of patient-driven engagement to keep exercising.

### The importance of rehabilitation in PD

Exercise and physical therapy are known to improve motor and non-motor symptoms of PD, which is highlighted by several meta-analyses underline the importance and positive effects of physiotherapy on motor symptoms. Significant improvements of gait, functional mobility, balance, motor symptoms, increased muscle strength, and reduced falls in PD patients who received physical therapy in short- and long-term follow-ups ranging from 1 to 12 months have been shown^14–17^. Another meta-analysis, covering 20 randomized controlled trials, showed significant improvement of non-motor symptoms in PD including neurocognitive manifestations, mood disorders, sleep disorders, and fatigue by physical therapy^18^. Trials investigating alternative sports, such as Tai Chi and dancing, revealed similar benefits on motor- and non-motor symptoms of PD^19–22^.

Next to physiological, structural, and clinical changes through exercise, physical therapy might even hold the potential to change the course of disease by activating neuroprotective mechanisms^23–25^. Based on animal studies, both neuroprotective and disease-modifying effects seem to be induced by exercise. For example, brain neurotrophic factors can induce neuronal protection and repair mechanisms in the dopaminergic system, which, in turn, increases angiogenesis and functional compensation mechanisms via glutamatergic and serotonergic circuits^26–28^. In PD patients, it has been shown that an exercise-induced increase of neurotrophic factors is associated with increased gray matter volume and symptomatic changes^16,27,29^.

Different types of physical exercise show different effects on motor and non-motor symptoms. This is, for example, reflected by treadmill studies leading to improvements mainly of gait, compared to exergaming studies, which have been associated with changes of balance and quality of life^30^. In view of the different PD types and degrees of severity, subjectively varying degrees of disturbing symptoms, as well as personal preferences, it becomes clear that one single type of training can hardly meet all requirements of all PD patients^28,31^. Therefore, large-scale comparative studies to recommend a single, “best” sport for motor and non-motor symptoms of PD and different PD subgroups are still needed^31–36^. In the meantime, an individual approach with choice of the sport in consideration of the mentioned aspects is necessary^31,35^. In COVID-19 pandemic times there is an additional argument for physical rehabilitation and exercising in PD. A worsening of PD symptoms and possible higher susceptibility to viral infections including COVID-19-infection seems almost inevitable once physiotherapy is stopped for a longer period. This is also gaining in relevance during these times as physical exercise reduces the risk of upper respiratory tract viral infections and the associated mortality in a dose-dependent manner. Here, moderate intensity exercise (in contrast to high intensity) has the best cell- and cytokine-based effect on the immune system and should be kept in mind when it comes to trainings plans^37^. Summarizing, an overwhelming body of evidence proves that multi-faceted physical therapy might offer preventive and moderating effects on the course of PD, is a vital mainstay of treatment covering all aspects of PD symptoms and might reduce susceptibility to viral infections and associated mortality.

### Impact of the COVID-19 lockdown on PD patients

COVID-19 and PD appear to interact with each other: there is evidence of an increased risk of COVID-19-associated mortality in PD as high as 20–40%^1^ compared to a 7–19% mortality risk of the general population. On the other hand, as with other infections, PD symptoms aggravate during COVID-19 infection. As PD patients are an elderly, often chronically ill patient group they are already within the high-risk groups for COVID-associated morbidity and mortality^1^. Furthermore, there is a potential link between COVID-19 and PD, which has shown high titers of anti-coronavirus antibodies in the cerebrospinal fluid of PD patients compared to healthy controls and other types of neurological illnesses^5^. This seems highly relevant, as the aggregation-susceptible alpha-synuclein might be also implicated in the innate anti-viral immune response^5^. At present, only self-quarantining offers sufficient protection against the virus. While effective against infection, the concept of quarantine opposes the usual recommendations for PD patients of keeping physically active. Due to their reduced cognitive flexibility and their dependence on regular physical exercise, they are vulnerable to serious collateral damage from the COVID-19 pandemic on their physical and mental health^11^. The combination of psychological stress and reduced physical activity can lead to a deterioration in motor symptoms that may start a vicious circle of depressed mood, lack of motivation, and further decline of mobility^38,39^. Although not infected, 10–28% of PD patients reported a worsening of their symptoms due to restricted mobility during the lockdown^40–43^. Italian PD patients spontaneously lamented the lack of rehabilitation programs after the suspension of standard clinical visits and trials when the country was ravaged by the pandemic. An observational study on 100 PD patients identified a marked decrease in exercise during the COVID-19 lockdown and later reports of deterioration of PD symptoms in half the study population. The patients who continued with their exercise regimen suffered from significantly less subjective aggravation of their PD symptoms, although the difference in the motor part of the MDS-Unified Parkinson’s Disease Rating Scale (MDS-UPDRS III) score between the groups did not reach significance^42^. Another aspect to consider is the direct and indirect effects of the pandemic on the psychological state of PD patients. Up to 30% of Parkinson’s patients suffer from concomitant depressive symptoms and self-quarantining can increase symptoms of depression and anxiety^4,44,45^. It is particularly important to consider this area of non-motor symptoms of PD patients and to offer treatment and support.

### Recommendations for rehabilitation measures during the COVID-19 pandemic

The Centers for Disease Control and Prevention^46^ and the Centers for Medicare and Medicaid Services^47^ recommend to curb the spreading of COVID-19 and reschedule elective procedures and treatments. Healthcare professionals were asked to resort to telemedicine for all other, either time-sensitive or functionally disabling, indications. This led to a quick implementation of telemedicine to replace routine or emergency clinical visits with remote check-ups via telephone or internet to extend care into the patients’ homes^41^. While the chronic movement restrictions caused by PD do not meet the criteria for time-sensitive indications (except recharging of empty Deep Brain Stimulation-batteries), they qualify as functionally disabling^9^.

Continuing with adapted physical activity and rehabilitation is therefore strongly recommended with either on-site measures, including reduction of patient count, prohibition of escorts, symptoms screening, physical distancing, and personal protective equipment, or via the increasing opportunity of telemedicine and telerehabilitation^4,11,12,48–50^. Ideally, a regime of moderate-intensity exercise at home 5–7 days per week is recommended, wherever possible outside^51–53^.

#### Telerehabilitation

**Fig. 1:**
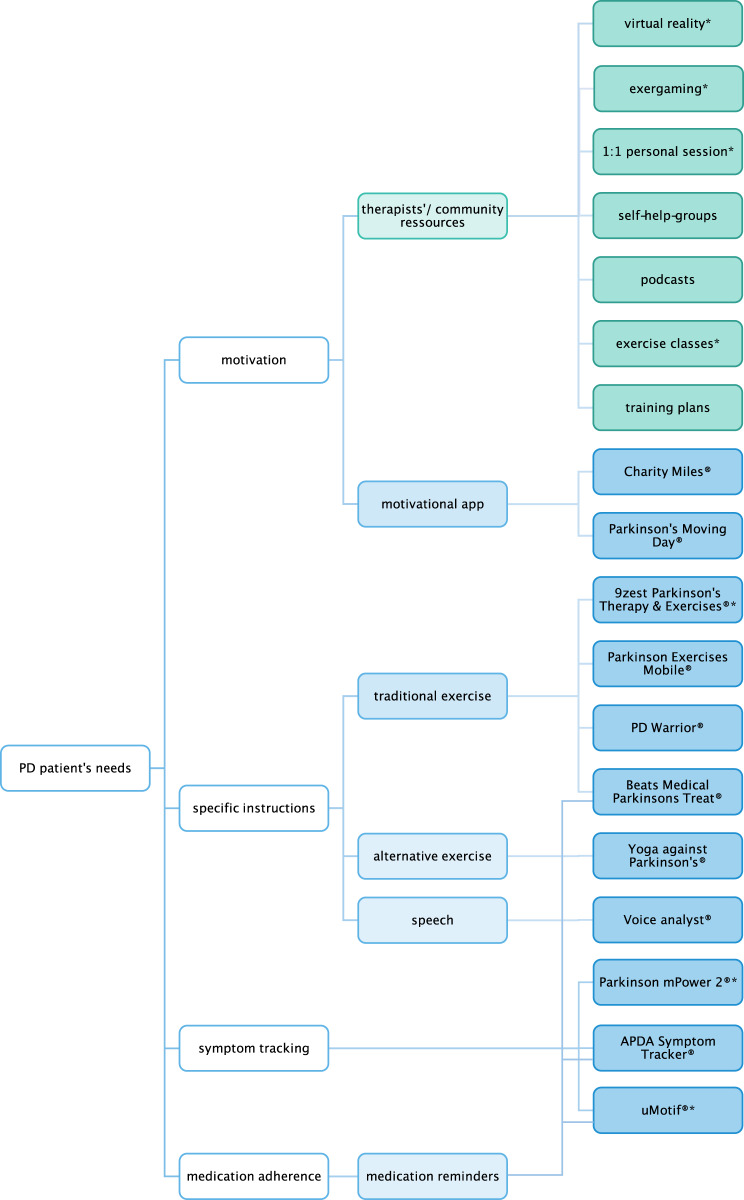
Decision tree for remote exercise-based treatment options. The figure shows the various needs of PD patients and their respective telemedical solution. White boxes: patient’s needs. Green boxes: online resources. Blue boxes: mobile applications. Virtual reality: patient-controlled avatar performs playful exercises to train balance, gait, or fine motor skills using motion sensors (either hand-held, body-mounted, or via a pressure-sensitive platform) and a headset or screen^76,77^. Exergaming: videogames demanding physical participation designed to improve motor skills^58,59^. 1:1 personal session: live therapeutic session with trainer or therapist via internet-based video calls^55–61^. Self-help groups and podcasts: motivational community resources on social media^85–89^. Exercise classes: web-based exercise options delivered by experts^72–75^. Training plans: downloadable plans for individual use^90–92^. Motivational apps: encouragement to stay active by donating to PD research (Charity Miles^104^, Parkinson’s Moving Day^105^). Traditional exercise: combination of motor, speech, and dexterity exercises (9zest Parkinson’s Therapy & Exercises^110^, Parkinson Exercises Mobile^111^, PD Warrior^112^, Beats Medical Parkinsons Treat^109^). Alternative exercise: yoga postures potentially beneficial for postural control and against rigidity (Yoga against Parkinson’s^113^). Speech: speech exercises for correction of hypophonia (Voice analyst^114^). Symptom-tracking: symptom tracking apps to monitor treatment response and optimize care by generating reports for discussion with physician and/ or physical therapist (Parkinson mPower 2^107^, APDA Symptom tracker^106^, uMotif^94,108^). Options with scientific evidence of feasibility and/ or effectiveness are indicated by an asterisk*.

Telerehabilitation comprises home-based, professionally guided training sessions accessed via telecommunication devices such as video calls either conducted in a life, one-on-one trainer-based fashion, or via pre-recorded classes, often combined with apps. It allows for professionally instructed training sessions without the risk of infection for neither the participant nor the trainer or therapist^54^. In addition to the advantages described above, telerehabilitation in PD patients is at least non-inferior to traditional rehabilitation in improving gait and balance as well as speech^55–61^. Telerehabilitation is recommended by the American Physical Therapy Association^62^, the Chartered Society of Physiotherapy^54^, the World Confederation for Physical Therapy, and the International Network of Physiotherapy Regulatory Authorities^63^. The successful implementation in other conditions such as stroke^64–67^, cerebellar ataxia^8^, muscular dystrophies^68^, hereditary spastic paraplegia^6^, and multiple sclerosis^69^ makes it a promising therapeutic possibility in PD. A recent study showed the readiness of PD patients to take part in telerehabilitation^70^. They offered a Zoom-based exercise program for newly diagnosed PD patients, which led to an unexpectedly high recruitment rate within weeks. In their ongoing study, they use up to four web-based individual training lessons to promote long-term changes to a higher level of physical activity^70^. In a randomized controlled trial, PD patients significantly improved their upper limb-mobility and functional mobility after a 3 month-telerehabilitation course while the control group’s balance and functional mobility deteriorated^71^. For an individual approach, an increasing number of physical therapists has started to offer remote physical therapy with the outbreak of the pandemic. Internet-based video calls connect the trainer and the patient at home. They then carry out the training session in the usual manner apart from adaptations made necessary by spatial requirements and equipment in the patient’s home^54^. To complement the one-on-one trainer-based fashion of telerehabilitation, video tutorials, and app support, delivered by experts are among emerging web-based exercise options (see Table 1 and Fig. 1)^11,72–75^.

**Table 1:** Overview of telerehabilitation methods.

	gait	rigidity	dexterity	balance/ posture	flexibility	speech	non-motor symptoms	medication adherence	symptom tracking	Advantage	Disadvantage
Virtual reality											
NintendoWii^®^, XboxKinect^®^, customized VR tools	✓			✓	✓					SE^74,75^	EN
Exergaming											
NintendoWii^®^	✓		✓	✓			✓			SE^113–123^	EN
Apps											
9zest Parkinson’s Therapy & Exercises^[®107^	✓	✓	✓	✓	✓	✓	✓			SE^98^	charges apply
ABF-gait app^®^, FOG-cue app^[®93^				✓						SE^93^	EN, ST
APDA Symptom Tracker^[®104^								✓	✓	SR	LE; mainly non-instructional
Beats Medical Parkinsons Treat^[®106^	✓		✓			✓		✓		wide variety of target symptoms	LE
Charity Miles^[®102^	✓									RF	LE; non-instructional
Parkinson Exercises Mobile^[®109^	✓			✓	✓		✓			wide variety of target symptoms	LE, charges apply
Parkinson mPower 2^[®105^									✓	SE^94,95^, SR	mainly non-instructional
Parkinson’s Moving Day^[®103^	✓									RF	LE; non-instructional
PD Warrior^[®131^	✓	✓	✓	✓						wide variety of target symptoms	LE
uMotif^[®92^								✓	✓	SE^132^	ST
Voice analyst^[®113^						✓				real-time analysis	LE, charges apply
Yoga against Parkinson’s^[®111^				✓	✓		✓			mind–body approach	LE

The main target areas of the respective method are indicated by a checkmark.

*EN* equipment needed, *LE* lack of scientific evidence, *RF* research funding, generates funding for future research, *SE* scientific evidence of feasibility and/ or effectiveness; see text/ citations for details, *SR* symptom reports, improves doctor–patient-communication by generating reports of symptoms to monitor treatment response and optimize care, *ST* only in the context of the study.

#### Virtual reality

A subspecialty of telerehabilitation based on VR exercises has shown promising results on motor and non-motor symptoms in PD and is increasingly recognized as a valuable alternative to traditional physiotherapy. Using motion sensors (either hand-held, body-mounted, or via a pressure-sensitive platform) and a VR headset or screen, VR exercises enable interaction with a computer-generated scenario in which a patient-controlled avatar performs playful exercises to train balance, gait, or fine motor skills. A systematic review covering eight trials with 263 patients comparing VR training with standard physical therapy found improved gait characteristics, balance, and quality of life, as well as satisfactory regime adherence, though the level of evidence was judged to be low due to methodological concerns^76^. Later, another randomized trial of 76 PD patients revealed significant improvement in balance in the intervention group after a 7-week VR-based balance training compared to traditional sensory integration balance training of the control group (see Table 1 and Fig. 1)^77^.

#### Motivational tools

A huge challenge associated with unsupervised activity is the lack of motivation to start and keep a regular exercise regime. Apathy in PD patients can further aggravate demotivation, though exercise itself reduces apathy and fatigue^18,21,78–81^. The World Health Organization (WHO) recommends measures to promote motivation not only at the professional level by health professionals but also at the community level (formation of training groups) and the personal level by promoting the patient’s rights of self-determination^82^. The patient’s autonomy has a major influence on motivation. Being able to choose the type, time, and place of training independently achieves better long-term adherence than fixed plans^83,84^. Telecommunications-based resources are available for both community-based and individual motivation. An online community of fellow patients and professionals supplies encouragement and support through self-help-groups on social media, networking podcasts, instructional videos, and follow-along exercise classes^85–89^. Exercise programs and instructions in the form of downloadable training plans are also available for individual use (see Table 1 and Fig. 1)^90–92^.

#### Apps

A variety of apps covering relaxation and meditation, motivation, symptom tracking, and exercises for balance, speech, gait, cognitive functions, and coordination are available^4,93^. Despite the increasing use, scientific evidence on apps for PD is still sparse. In the light of the increasing field, we present hereafter a selection of current smartphone applications, useful for PD during the COVID-19 pandemic (see Table 1 and Fig. 1). In a randomized controlled study, a 16-week implication of the “*Parkinson’s Tracker App uMotif*” was able to significantly improve medication adherence, satisfaction with the medical consultation, and non-motor symptoms^94^. One randomized controlled trial has shown some first positive effects of app use on gait in 40 PD patients: although both groups showed significant improvement of gait speed, only the intervention group who performed a 6-week, thrice-weekly gait training course guided by the “*ABF-gait app*” and the “*FOG-cue app*” improved postural control and quality of life compared to the active control group who performed the same gait exercises without app-support^95^. The app relies on two external motion-sensor units, which might limit the app’s implementation in real-world telerehabilitation. Several observational studies have already shown that mobile apps are feasible both for recording and registering symptoms in a highly reliably manner for scientific purposes and for training to improve motor functions in PD^96–99^. A recent study on 28 PD patients evaluated the App “*9zest Parkinson’s Therapy & Exercises*” and reported it to be both feasible and effective. After 3 months of training, functional lower extremity strength, mobility, and quality of life improved^100^. Other feasibility studies on software programs supplemented by wearable motion-sensing technology are currently on their way^101,102^. A randomized controlled study of 51 PD patients showed superior effectiveness of a 12-month multimodal telerehabilitation course, using individualized training plans, video tutorials, and app support with significant improvement of mobility compared to the physically active control group without telerehabilitation, in which the subjective mobility-related quality of life as measured by the Parkinson’s Disease Questionnaires-39 (PDQ 39) mobility domain scores even deteriorated during the study period^103^.

### Outlook of ongoing App projects

The mobile apps “*Charity Miles*” and “*Parkinson’s Moving Day*” encourage PD patients to stay active by donating to PD research according to their walking distances. This effectively serves two purposes in one: immediate benefit for the individual through exercise and long-term influence by funding research^104,105^. The *“APDA Symptom Tracker”* app by the American Parkinson Disease Association eases the increasingly vital remote care by letting PD patients record their symptoms to discuss their report with their neurologist at their next telehealth appointment^106^. “*Parkinson mPower 2*“*,* which has already been validated by an observational study, not only lets PD patients record their symptoms, but also gives them the opportunity to make their data available for scientific study purposes^96,97,107^. “*Parkinson Exercises Mobile,*” “*PD Warrior*^108^,” “*9zest Parkinson’s Therapy & Exercises,*” and “*Beats Medical Parkinsons Treat*” offer a combination of motor, speech, and dexterity exercises^109–112^. In the app “*Yoga against Parkinson’s,*” PD patients are guided through selected yoga postures that are considered beneficial for postural control and greater flexibility, as well as relaxation^113^. “*Voice analyst*” helps to correct hypophonia by analysing the speech of PD patients in real-time^114^.

#### Exergaming

For a more entertaining and therefore motivational approach, exergames, i.e., videogames demanding physical participation designed to improve motor skills, are increasingly recognized as a valuable alternative to traditional exercise. Two reviews of 7 and 22 studies on Nintendo Wii^™^ based exergaming interventions in patients with PD or the elderly, respectively, showed significant improvements in upper limb function and strength, balance and functional mobility, a reduction of falls, and an improvement of both neurocognitive abilities and psychosocial aspects^115,116^. In PD patients, two randomized controlled trials revealed a significant improvement of balance after an 8- or 12-week exergaming training regime^117,118^. Exergaming proved to be at least as effective in improving functional mobility and gait as traditional balance exercising, physiotherapy, or bicycle training in a randomized trial of 32 PD patients^119^. It furthermore significantly improved upper limb function in two series of PD patients^120,121^. Although a randomized trial failed to reproduce the same significant results of dexterity improvement after a 12-week exergaming course, it still showed increased speed in the nine-hole peg test but at the cost of accuracy. This was explained as a direct effect of those exergames which prioritize speed training while neglecting precision^122^. Even cognitive-motor dual-tasking functions improved significantly after exergaming sessions compared to controls^123^. A systematic review on exergaming in PD summarized found exergaming to be equally effective, as well as practical and safe, as traditional rehabilitation^58^. An elaborate recent study showed the feasibility of guided home exercising in PD patients, using app support and motivating exergaming experience. Within this large randomized study, 65 PD patients who underwent aerobic training for 6 months showed significant motoric improvement in the motor part of the MDS-UPDRS III compared to the control group who only performed regular stretching^59^.

### Creative self-initiative on the part of the patients: A case presentation

An extraordinary example of self-engagement as presented here can be highly inspirational to other patients who cannot follow their conventional training schedule and routines during the COVID-19 pandemic. In February 2019, a 69-year-old woman presented in our outpatient clinic with a tremor-dominant PD, with symptoms starting in 2004. Neurological examination revealed a left dominant rest and postural tremor, left dominant mild bradykinesia, moderate axial and limb rigidity, a typical hypokinetic gait, mild dyskinesia, and a slightly stooped posture. She complained about neck pain and sleeping problems. Her daily PD medication included levodopa 200 mg, rotigotine 8 mg, and rasagiline 1 mg.

Without earlier climbing experience, she took part in a 12-week guided climbing course for patients with PD once a week for 90 min, where she learned top-rope climbing and belaying skills. She strongly benefitted from the course: her motor and non-motor symptoms improved, for example, and the neck pain disappeared.

When the climbing hall closed during the COVID-19 pandemic, her motor and non-motor symptoms deteriorated, and her neck pain reoccurred. In the week after the lockdown, she started climbing again, using an extra-long rope ladder that she attached to a branch of a tree in her garden for top-rope climbing. Secured by her husband, she continues climbing up the rope ladder at least 1–2 times per week.

When she started with her garden climbing experience in April 2020, she could climb the first 3–4 rungs and made progress within 2 weeks. Since then, she has been regularly climbing the whole ladder with 14 rungs 3–4 times without interruption for 7 months now (see Video, Supplemental Digital Content 1, which shows the patient climbing her garden rope ladder).

She can still perfectly control her neck pain with this exercise. It disappears during or after the training and does not return for 4–6 days. She also reported that during the regular climbing exercises in the garden, her sleep quality, her sense of body balance, and her left-sided weakness improved. Even if we perceive this type of exercise to be a great innovation and to have high potential in PD treatment, it is essential to readers to not mistake the exercise (and extreme training method) described above as a general recommendation for PD patients. This patient’s individual training method only worked well because she and her husband had previously gone through special climbing training sessions and had carefully examined the climbing mount and the tree for stability. We would like to emphasize the importance of performing physical exercise for PD patients only in a safe environment as osteoporosis is pandemic in PD and fractures due to falls must be avoided at any time. Patients and caregivers must consider this aspect when selecting and planning sports giving preference to low-risk sports with adequate (remote or personal) supervision.

## Conclusion

Discontinuity in exercise-based therapy due to the COVID-19 pandemic has already had a detrimental effect on motor and non-motor symptoms, as well as on the wellbeing of PD patients. Most of the consequences are not yet visible and will only show later in long-term after-effects. Counterstrategies are based primarily on implementing comprehensive telerehabilitation programs, as they have shown great potential in the long-term remote care and support for PD patients. All the articles discussed earlier proposed the implementation of telehealth or telerehabilitation during the ongoing COVID-19 pandemic as an alternative to conventional physical therapy and allied health since internet-based technology cuts the risks of infection through personal contact. This enables carers and patients to continue with the most important non-pharmaceutical therapy principle in the treatment of PD even in pandemic and lockdown times. A growing body of evidence suggests that telemedicine and telerehabilitation could be as useful as treatments concerning functional outcomes^124^. Other benefits of remotely supplied treatment are the reduced costs and improved convenience by cutting travel expenses and burden^51–53^.

Some aspects should however be specifically addressed in future studies. While patients receiving telemedicine are satisfied with the provided service^124^, those who do not have access to the technology or the necessary knowledge and confidence to use the resources must have a chance to receive treatment of comparable quality.

### Future outlook

The COVID-19 pandemic, as terrible as it undoubtedly is, holds the potential for an unplanned but an unavoidable test phase for telemedicine. This raises the question, if telemedicine can and should support, and partly substitute, traditional personal medicine in view of the advantages described above even after the pandemic.

Despite the general patient satisfaction with telemedicine, prior research and recent experience of COVID-19 lockdown phases show that patients sorely miss the personal contact with doctors and therapists. Both sides are not willing to completely abandon personal care in future^11,41,43,125^. Despite comparable quality of remote appointments, they cannot provide a perfect substitute for traditional in-person visits with a direct physical examination^126–128^.

On the other hand, telerehabilitation offers the best possible treatment to patients in areas without easily accessible neurologists or therapists and also patients who live in the vicinity of therapy facilities should get the opportunity to supplement their standard treatment with telerehabilitation. It seems to be an inexpensive alternative to conventional physiotherapy and should be promoted as one way of high-quality care for patients without routine access to healthcare institutions^129,130^. However, long-term cost-effectiveness calculations are not available yet and should be investigated in future research^51–53^. From today’s perspective, it is very likely that an individual “hybrid model“ of traditional in-person medicine and some form of telerehabilitation will prevail.

## Methods

### Narrative Review

We performed a literature search in the PubMed database using the search criteria “COVID-19”, “Parkinson Disease”, “telerehabilitation”, “physiotherapy”, “exercise”, “virtual reality”, “exergaming”, “application”, screened the references of relevant articles for additional relevant publications, and searched official regulatory website of health authorities for recommendation on telemedicine. Relevant cell phone applications were identified in the app stores “App Store (iOS)”^131^ and “Google Play Store”^132^^,133^ using the search term “Parkinson Disease”.

### Case report

After having completed a guided climbing course as a part of a clinical study (approved by the ethical committee of the Medical University of Vienna No: 1369/2017), the patient decided to continue her climbing training at home. The patient volunteered the information about her individual climbing training during a routine visit and spontaneously provided the Supplementary Video 1. The patient provided written informed consent to participate in this case report. The authors affirm that the human participants provided written informed consent for publication of the video in Supplementary Video 1.

### Reporting Summary

Further information on research design is available in the Nature Research Reporting Summary linked to this article.

## Supplementary information

Supplementary Video 1

Supplementary Information

reporting summary

## Data Availability

Data are available on request from the authors.

## Code availability

No software or custom code was used for data collection or analysis.
