# Supplementary material for: How COVID-19 will boost remote exercise-based treatment in Parkinson’s disease: a narrative review
Source: NPJ Parkinsons Dis. 2021 Mar 8;7:25. doi: 10.1038/s41531-021-00160-3 (PMC7940641; doi:10.1038/s41531-021-00160-3)
Supplement: Supplementary file 2 — Supplementary Information [file 41531_2021_160_MOESM2_ESM.pdf]

Supplementary Video legend

Supplementary Video 1: Top-rope climbing on a rope-ladder. The patient (left) is shown climbing a rope ladder that has been fixed to the upmost branches of a tree in the patient's own garden. The patient's husband (right) can be seen securing the patient by holding a rope fixed to the patient's climbing harness and his own harness.
